# Eight Years of Follow-Up of Rituximab in Pemphigus Vulgaris and Foliaceus at a Single Center: Assessing Efficacy and Safety in Light of Several Factors

**DOI:** 10.3390/jcm14207318

**Published:** 2025-10-16

**Authors:** Konrad Szymanski, Cezary Kowalewski, Irena Walecka, Katarzyna Wozniak

**Affiliations:** Department of Dermatology, National Medical Institute of the Ministry of the Interior and Administration, 02-507 Warsaw, Poland; kszyma@gmail.com (K.S.); cezary.kowalewski@pimmswia.gov.pl (C.K.); irena.walecka@pimmswia.gov.pl (I.W.)

**Keywords:** rituximab, pemphigus vulgaris, pemphigus foliaceus, BMI, PDAI, COVID-19

## Abstract

**Background/Objectives**: Pemphigus vulgaris (PV) and foliaceus (PF) are autoimmune blistering diseases mediated by IgG antibodies directed against desmogleins 1 and 3 and are still considered life-threatening disorders. In recent years, rituximab has been shown to be very effective, especially in PV and mainly in short follow-ups. The role of rituximab in achieving long-lasting complete clinical remission (cCR) in pemphigus still needs to be determined. Therefore, the aim of our study was to assess the efficacy, measured by achieving long-lasting cCR, and safety of rituximab in both PV and PF over an 8-year follow-up in light of several factors (body mass index—BMI, severity of disease—PDAI, age, gender, disease duration, COVID-19 period). **Methods**: In total, 28 patients with pemphigus were treated with rituximab and followed-up at one center. The entire analysis was performed using statistical methods. **Results**: Long-lasting cCR was achieved in 5 out of 6 patients (83%) with PF and 10 of 22 (45.5%) patients with PV. Univariate and multivariate analysis disclosed that studied factors did not statistically correlated with achieving long-lasting cCR. Among studied patients, few developed side effects, mainly urinary tract infection; one patient had sepsis, and one patient died. **Conclusions**: This study has demonstrated that rituximab is highly effective in PF and quite effective in PV over an 8-year follow-up in relation to independently studied factors. Moreover, the COVID-19 pandemic was not a negative factor influencing cCR achievement since 82% of patients treated with rituximab during that time still achieved cCR.

## 1. Introduction

Pemphigus is a group of autoimmune blistering diseases of the skin and mucous membranes mediated by IgG antibodies directed against desmogleins (1 and 3)—antigens responsible for cell–cell adhesion [1]. Before the era of corticosteroids, the course of the disease was progressive, leading to death within about 2 years of diagnosis [2]. The introduction of prednisone and immunosuppressive drugs has significantly improved the prognosis for pemphigus, but the disease is still considered life-threatening due to the numerous side effects of these drugs. In recent years, the efficacy and safety of rituximab have been repeatedly demonstrated, mainly in the case of pemphigus vulgaris (PV) and in short-term follow-ups [2,3,4]. However, the role of rituximab in achieving long-term complete clinical remission (cCR) still needs to be determined. Furthermore, data on the role of rituximab in pemphigus foliaceus (PF) in both short-term and long-term follow-ups are very scarce.

Therefore, the purpose of this study was to analyze the clinical response to rituximab and the role of several factors (age, gender, body mass index, severity of the disease, duration of the disease before rituximab infusion, and COVID-19 pandemic) in the efficacy and safety of rituximab in both PV and PF in short- and long-term follow-ups.

## 2. Materials and Methods

Patient characteristics is depicted in Table 1.

### 2.1. Patient Characteristics

In total, 28 patients with pemphigus were treated with rituximab and followed-up at one center between 2017 and 2025. The diagnosis of pemphigus was established on the basis of clinical features and immunopathological tests: direct immunofluorescence (DIF), indirect immunofluorescence (IIF) and ELISA in all patients. All patients presented IgG deposits in the intercellular spaces of the skin or oral mucosa according to DIF and circulating IgG pemphigus antibodies according to IIF. Additionally, the reactivity of circulating antibodies with desmogelin 3 and/or 1 was confirmed using 6-antigen dermatological profile ELISA (Euroimmune) in all patients. No other antigens were recognized by circulating antibodies in the studied patients.

Out of 28 patients, 22 were diagnosed with PV and 6 with PF. In the PV cohort, the median age was 51.5 years (range: 30–68), and women accounted for 59.6% of this group. The duration of PV before rituximab treatment varied from 3 to 180 months, with a median of 28 months.

In the PF cohort, the median age was 54.0 years (range: 40–67) and 67% were female. The history of PF before rituximab treatment ranged from 7 to 108 months (median 60.0).

### 2.2. Characteristics of Patients’ Treatment Before Rituximab Administration

Before rituximab administration, all patients but one were treated with prednisone in different doses. In 8 (PV–7, PF–1) of 28 patients (28.6%), prednisone was used as a monotherapy; adjuvants were never used in these patients. The remaining 20 patients (71.4%) were treated with prednisone plus any adjuvants (dapsone, azathioprine, cyclophosphamide, IVIG, mycophenolate mofetil, tetracyclinum) in various periods of their illness.

### 2.3. Disease Severity at the Time of Rituximab Administration

Disease severity was assessed using a questionnaire named the Pemphigus Disease Activity Index (PDAI) [5]. In the PV group, the PDAI ranged from 2 to 66 (med 15.9). Only two patients in this group had PV limited to the oral mucosa. The other 20 PV patients presented lesions located on different parts of the body including the genital area (1 female patient) and scalp (5 female patients and 1 male). In turn, in the PF group, the PDAI ranged from 8 to 39 (med 22.5). Thus, the patients included in the study suffered from mild to severe pemphigus.

### 2.4. Body Mass Index at the Time of Rituximab Administration

In the PV group, body mass index (BMI) ranged from 19.1 to 36.9 (med 31.4). BMI was above 25 in 72.7% of these patients. In the PF group, BMI ranged from 21.1 to 34.6 (med 28.8) and was above 25 in 83.3% of those patients.

### 2.5. Rituximab Administration in the Period of COVID-19 (Between January 2020 and December 2022)

Out of 28 patients analyzed in the current study, 17 (60.7%) were treated with rituximab during the COVID-19 pandemic. Fourteen were treated once, whereas the three patients were treated twice, 2 years apart.

### 2.6. Treatment Protocol

Patients included in this study were qualified for treatment with rituximab due to corticosteroid dependence, recurrent disease, or contraindications to immunosuppressants. Informed and written consent was obtained from all patients. Patients with paraneoplastic pemphigus were excluded from the study, as were patients with PV and PF who disappeared from the follow-up immediately after rituximab administration.

Overall, the group of patients included in this study was homogenous in terms of treatment protocol. This means that when the decision was made to use rituximab, prednisone was reduced to a dose of 0.5 mg/kg body weight and all adjuvants were discontinued in all patients one month prior to rituximab infusion. Rituximab was administered at a dose of 2 × 1 g two weeks apart in all patients. Subsequently, prednisone was gradually tapered within 6–8 months to 5 mg/day, then to every second day and eventually discontinued.

### 2.7. Statistical Analysis

The baseline characteristics of patients were described using median, minimum, and maximum values. The chi-squared (χ^2^) test was applied to compare categorical variables, while the unpaired two-tailed t-test or the Mann–Whitney test was utilized for comparing quantitative variables. To identify the factors influencing remission, logistic regression analyses were performed. A *p*-value of less than 0.05 was considered statistically significant. Data analysis was conducted using Jamovi software version 2.6 [6].

This study was approved by the local institutional bioethical committee.

## 3. Results

**Figure 1:**
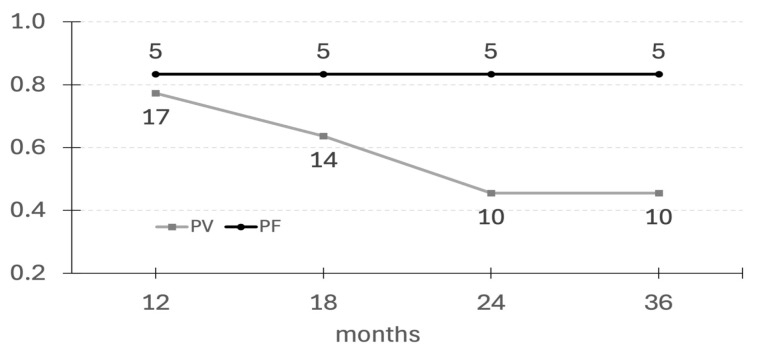
The number of patients achieving long-lasting cCR after first infusion of rituximab. Legend: cCR—complete clinical remission, PV—pemphigus vulgaris, and PF—pemphigus foliaceus.

The results are presented in Table 2, Table 3 and Table 4 and Figure 1.

### 3.1. The Efficacy of a Single Cycle of Rituximab

Time to cCR achievement ranged from 1 to 8 months (med 1.75) in the PV group, whereas in the PF group, it was between 1 and 9 months (med 5.5).

The proportion of patients remaining in cCR was observed over time.

cCR lasting at least 12 months was observed in 17 (77.3%) patients with PV and 5 (83.3%) patients with PF.

cCR lasting at least 18 months was observed in 14 (63.6%) patients with PV and 5 (83.3%) patients with PF.

cCR lasting at least 24 months was observed in 10 (45.5%) patients with PV and 5 (83.3%) patients with PF.

cCR lasting at least 36 months was observed in 10 (45.5%) patients with PV and 5 (83.3%) patients with PF. In this group, there were four patients (PV–3, PF–1) who had been in cCR for 3 years after one cycle of rituximab infusion, seven patients (PV–5, PF–2) who received rituximab 4 years ago, and four patients (PV–2, PF–2) who received rituximab more than 5 years ago.

In summary, among the 28 studied patients, 15 (53.5%) never experienced pemphigus relapse after a single infusion of rituximab in the studied time period. Eleven patients (39%) experienced at least one relapse of the disease. One patient (with PF) was lost to follow-up.

### 3.2. Relapses After Single Cycle of Rituximab

Relapses after a single cycle of rituximab infusion were observed in ten PV patients and one PF patient. Relapses usually occurred 18–24 months after rituximab infusion. All of these patients were treated repeatedly with rituximab from 1 to 3 times in the studied time frame, whereas one PV patient was additionally treated with intravenous immunoglobulins (IVIGs). Remission after each infusion of rituximab varied from 18 to 48 months.

### 3.3. Association Between Achieving Long-Term cCR and Gender, Age, PDAI, BMI, and Disease Duration Prior to Rituximab Administration

Among patients who achieved long-term remission, 63.6% of those with PV and 80% of those with PF had a BMI above 25; however, this did not significantly impact the chance of achieving remission, with *p*-values of 0.618 for the PV group and 0.215 for the PF group.

In univariate (Table 3) and multivariate (Table 4) logistic regression, we found no associations between gender, BMI, the PDAI, age during therapy, or disease duration prior to rituximab administration.

### 3.4. Adverse Effects Associated with Rituximab (Depicted in Table 2 and Figure 2)

**Figure 2:**
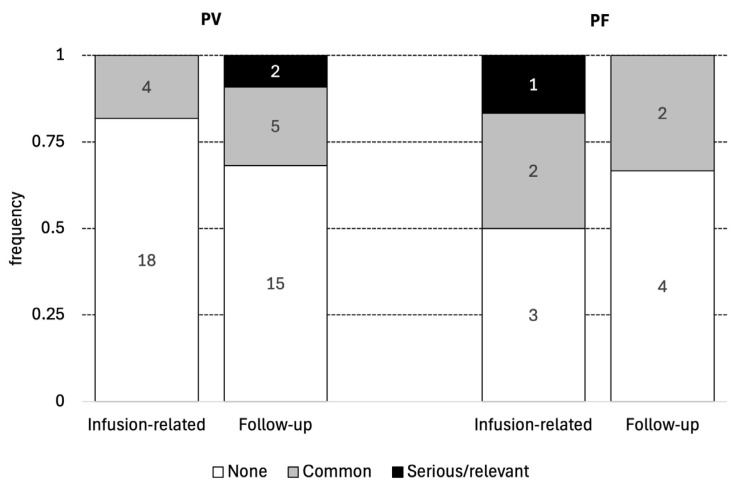
Adverse effects associated with rituximab administration.

Adverse effects related to drug infusion were observed in six patients: four with PV (18.2%) and two with PF (33.3%). None of the adverse effects were serious; however, sore throat, hoarseness, dry mouth, runny nose, watery eyes, nausea, and chills were observed. Joint pain, weakness, or fever developed immediately after an infusion or the next day. All symptoms were transient and resolved after reducing the infusion rate or administering antihistamines or paracetamol (see Table 2 and Figure 2).

Among the distant side effects (during follow-up), the most frequent were infections of the urinary tract. They occurred in six cases, of which four were uncomplicated and two occurred in PV male patients (at weeks 3 and 13 after rituximab infusion). One other PF male patient developed sepsis caused by *Staphylococcus aureus* at six months of follow-up, from which he recovered after antibiotic therapy. Moreover, one PF female patient developed an upper respiratory tract infection after 3 months, but this was successfully treated with antibiotics. Another PV male patient had morning hand tremors and pain in the knees and elbows at 5 months after rituximab infusion. One more PV male patient developed aseptic necrosis of the femoral head, requiring its replacement, after 12 months (see Table 2 and Figure 2).

Among the 28 studied patients, only 1 female patient died. She was a 76-year-old and suffered from severe relapsing PV. Two years after the third rituximab infusion, she became immobilized due to knee replacement surgery. At that time, pemphigus relapsed in the area of surgery, with subsequent spreading of blisters. For this reason, prednisone was reintroduced. Additionally, she suffered from diabetes, hypertension, and significant obesity. Therefore, it is likely that the fatal outcome was the result of her complex health condition and was not associated with rituximab. During treatment and the entire follow-up, no patient developed tumors.

### 3.5. Outcomes in Patients Treated with Rituximab During the COVID-19 Pandemic

Among 17 patients who were treated with rituximab during the COVID-19 pandemic, 14 of them (PV–11, PF–3) were treated one time and they remained in cCR, whereas the other 3 patients (PV–2, PF–1) experienced relapsed pemphigus 2 years later and required a second rituximab administration.

In terms of side effects, 4 of 17 patients developed mild-to-serious side effects: 1 PV female patient had candidiasis of all fingers of both hands, another (PV male) had aseptic necrosis of the femoral head with subsequent replacement, and another PV male patient developed myocardial infarction and venous thrombosis, however survived. Another female patient underwent knee replacement surgery, which was complicated, and the patient eventually died. All of these medical events occurred between 12 and 24 months after rituximab administration.

## 4. Discussion

For decades, prednisone has been the mainstay of treatment for pemphigus, used as monotherapy in milder cases or in combination with adjuvants in more severe cases [7]. However, high doses of these drugs are required for achieving long-term remission and usually lead to serious side effects with subsequent health devastation, time away from professional work, and eventually to a major reduction in quality of life [8].

In recent years, rituximab has been shown to be highly effective and safe in pemphigus [9]. Initially, it was reserved for patients with PV who were resistant to standard immunosuppressive drugs or had contraindications to their use or was used as an adjunct to conventional therapy. However, it turned out that such poligramasia is responsible for serious side effects and a risk of death in patients with PV [10].

Almost 20 years of experience and excellent results with rituximab in PV have led to its indications being extended to milder forms of the disease [11]. However, data on its role in PF are still scarce, especially in long-term follow-up. This is because PF is considered to be a milder subset of pemphigus, not requiring such aggressive immunosuppressive therapy like PV.

A study conducted several years ago by a French group showed that combining rituximab with low-dose prednisone (0.5 mg/kg) administered as short-term therapy was effective and safe in PV [12]. The authors showed that at month 24, 89% of patients were in complete remission and off therapy. This encouraged us to use that regimen in our patients in 2017.

In this article, we present the results of an 8-year observation study on the use of rituximab in 28 patients with pemphigus at a single center. This is probably the one of the longest observation periods for patients with pemphigus treated with rituximab in one center reported in the literature. An important factor of this study is the homogeneous group of analyzed patients in terms of therapeutic protocol, which contained rituximab at a dose of 2 × 1 g and prednisone at an initial dose of 0.5 mg/kg. All patients discontinued prednisone between 6 and 8 months from the beginning of the study. In 20 of the 28 patients, rituximab was a second-line treatment, while in the remaining 8 patients (PV–7, PF–1) (28.6%) it was a first-line treatment.

Our study found that a single infusion of rituximab led to cCR in 77.3% of patients with PV and 83.3% patients with PF in a 12-month follow-up. This observation is in line with other studies and most likely is the effect of the mechanism of action of rituximab, i.e., the inhibition of pemphigus antibody production for about 10–12 months with subsequent repopulation of CD20 cells [12,13,14].

Our study showed a significantly variable response to a single infusion of rituximab in patients with PV and PF. Interestingly, in the PF group, the percentage of patients remaining in cCR was stable during follow-up, 83% of patients, whereas in the PV group the percentage of patients remaining in cCR decreased steadily up to 36 months of observation. cCR lasting more than 36 months without any additional maintenance drugs was achieved in 53% of patients with both subtypes of pemphigus. It is worth underlining that if patients remained in cCR for at least 36 months, they had a good chance of maintaining long-term cCR since in this group there were 11 patients (39% of all) who had been in cCR off therapy for more than 4–5 years. This raises the question of whether these patients can be considered cured of pemphigus. This question is particularly relevant for patients with PF since all of them but one remained in long-lasting cCR after a single cycle of rituximab. The reason for that promising outcome is not clear, but it is likely that PF mediated by antibodies directed to one antigen (dsg 1) is easier to manage than PV, which is mediated by antibodies to both dsg 1 and 3, causing its pathogenesis to be much more complex. It is also known that PF patients usually require less aggressive immunosuppressive therapy than PV patients to achieve CR. This original observation in the PF group was not published before, but it may be an encouraging factor for the use of rituximab as first-line therapy in PF. However, further studies on large, uniform cohorts are needed to confirm this observation, especially as other authors have presented divergent results in single or small groups of patients. For example, the results of Nosrati et al. revealed that relapses occurred in 49% of their PF patients after a mean of 18 months [15]. In turn, the study by Shah et al. found that relapses after a single infusion of rituximab occurred in 35.5% of PF patients after a mean duration of 21.4 months [16]. Palacios-Álvarez et al. reported relapses in six patients with PF (50%) after a median of 12 months. The median age of patients in their cohort was very similar to ours, whereas the median disease duration before rituximab administration was significantly shorter than ours (46 months vs. 60 months) [17]. The authors suggested that CR rates in PF might be lower than expected if the disease is severe with a longer duration. Our findings contradict this explanation. On the other hand, de Sena Nogueira Maehara et al. revealed that 83% of their PF patients obtained remission after rituximab: six complete and four partial remission in short-term observation and an off-therapy outcome in 50% of patients after a mean of 64 weeks [18].

Some authors suggest that rapid administration of rituximab in pemphigus promotes the achievement of long-term cCR [19,20,21]. Our study is in opposition to previous ones, since during the 8-year follow-up, we found no correlation between rapid administration of rituximab and long-lasting cCR. It is worth underlining that in our cohort, the median duration of the disease before rituximab treatment was 2 times longer in the group of PF than in PV. Despite this, all but one PF patient never relapsed after the first administration of rituximab and are still in cCR. Moreover, in our group, the longest period of cCR, lasting 8 years, was achieved by a patient who had suffered from recurrent pemphigus for 14 years prior to treatment with rituximab. Interestingly, patients with PF needed on average three times longer (median 5.5 months) to achieve cCR than patients with PV (median 1.75 months). In previous studies, the time to achieve cCR varied and was likely related to the varying severity of skin lesions or the retrospective nature of the study [15,22,23].

Relapses after rituximab treatment in pemphigus are common. In the current study, they occurred in 47% of patients, mainly with PV, after the first rituximab administration. These patients were treated repeatedly with rituximab and one patient with IVIGs. The recurrence rate varies across previous studies [14,24]. This is due to different observational time frames and different treatment modes in individual studies. However, the common observation for the majority of studies is that relapses occurred usually between 12 and 24 months after rituximab administration. Remissions after each infusion of rituximab in the current study varied from 18 to 48 months.

It is widely accepted that one of the reasons for the ineffectiveness of rituximab in pemphigus is the production of human anti-chimeric antibodies (HACAD) against rituximab which are responsible for incomplete B-cell depletion and the lack of therapeutic success [25,26]. This phenomenon was observed in lupus erythematosus, immune thrombocytopenic purpura, and pemphigus [27,28,29]. Another reason for relapses after rituximab observed in individual pemphigus patients may be the presence of long-lived plasma cells which have the potential to produce pathogenic pemphigus antibodies [30]. Since they are “hidden” in the secondary lymphatic organs (lymph nodes, spleen), they are poorly accessible to rituximab [30]. This phenomenon occurs if the disease has been present for many years. Therefore, it was suggested that patients with pemphigus should be treated with rituximab as quickly as possible after the diagnosis has been made to avoid the development of the long-lived plasma cells [31]. The authors suggested that the longer the pemphigus duration, the lower the chance for long-lasting cCR after rituximab therapy.

The analysis in our cohort referred to two groups: patients who suffered from pemphigus for several years and received rituximab as a second- or third-line therapy and patients who received rituximab as a first-line therapy immediately after the diagnosis had been made. In univariate and multivariate logistic regression, we found no association between disease duration prior to rituximab administration and achieving long-lasting cCR in our 8-year follow up.

Other immunological factors hindering the achievement of long-term cCR in pemphigus after rituximab administration are still under discussion. We have analyzed the impact of age, sex, BMI > 25, and the PDAI > 35 on achieving long-lasting cCR (8-year follow-up) and we found no such association in univariate and multivariate logistic regression (see Table 3 and Table 4). Similarly, the study by Lunardon et al. also showed no relationship between age or sex and achieving cCR in a follow-up no longer than 28 months [19]. In turn, the studies by Mignard et al. showed that the PDAI is a negative risk factor, since patients with pemphigus relapse had a 2-fold higher PDAI than those without relapses. However, this observation referred to a 12-month follow-up [32]. In some articles, an elevated BMI, particularly equal or greater than 35, was suggested to be a risk factor for pemphigus relapse [14]. A recent observation by Aryanian et al. showed that obese males with pemphigus are more resistant to rituximab and prone to relapses [33]. In our cohort, more than 70% of patients had a BMI over the normal range, but this factor did not statistically significantly impact the chance of achieving long-lasting cCR. However, when analyzing individual patients from our group who experienced more than one relapse during the 8-year observation period, it turned out that these were mainly women with severe obesity (BMI > 30). Therefore, further studies are needed to establish whether BMI indeed plays a role in achieving long-lasting cCR.

An important factor to consider when treating with rituximab is the advanced age of patients, as this may affect its efficacy and safety. The study by Kushner et al. showed that an age of 65 or older was significantly associated with CR [14]. The authors suggested that the weakened immune system in elderly people may facilitate easier remission. In contrast, we found no statistically significant correlation between achieving long-lasting cCR and the patients’ age at the time of rituximab administration.

There are two main regiments for rituximab administration in pemphigus, 2 × 1 g two weeks apart in the rheumatology regimen and 4 × 375 mg/m^2^ one week apart in the hematologic regimen. In the literature, the rheumatological regimen is much more common in pemphigus. All our patients were treated according to that regimen, and thus the cohort is uniform for the analysis. However, to date, it has not been proven which therapeutic regimen is more effective. It seems that the hematologic regimen better reflects the amount of drug administered per body weight, its penetration, and consequently remission duration [23]. Further studies considering BMI and the mode of administration of rituximab are needed to determine their real significance in achieving long-term clinical remission in pemphigus.

With regard to the side effects of rituximab, our study revealed adverse events that had been described previously [34]. Uncomplicated urinary tract infections were the most common and occurred in six cases (21% of total) at various times after administration of rituximab; one other patient had sepsis at 6 months of administration of rituximab and fortunately survived. An unexpected adverse event was aseptic necrosis of the femoral head in a young man. This appeared at 12 months after rituximab administration and ended with its replacement. As the patient had a long history of treatment with high doses of prednisone (1–1.5 mg/kg), this complication was most likely related to prednisone rather than rituximab. Nevertheless, this event should be kept in mind during treatment with rituximab since it was also published by Yamagami et al. [35]. One of our patients died during follow-up; however, this was not related to rituximab administration. In general, rituximab appeared to be safe in our cohort and we believe that this was the result of a significant reduction in prednisone dose and complete withdrawal of immunosuppressive drugs prior to administration of rituximab.

Out of 28 patients analyzed in the current study, 17 (60.7%) were treated with rituximab in the period of the COVID-19 pandemic, caused by a virus affecting the immune system [36]. It is known that rituximab lowers human immunity for approximately 12 months and theoretically promotes the development of serious infections. On the other hand, high doses of prednisone and/or adjuvants required for stopping pemphigus are also responsible for numerous side effects. Eventually, our patients who developed new or recurrent pemphigus during the COVID-19 pandemic underwent rituximab therapy after a thorough analysis of their general health condition and weighing up the benefits and risks associated with this treatment. The majority of them (14 of 17 patients) were treated once in the pandemic and remain in cCR to date. Only three patients in this group relapsed 2 years later and they required a second rituximab administration. During this time, two patients developed serious medical incidents, i.e., aseptic necrosis of the femoral head (mentioned above) and myocardial infarction and venous thrombosis, which occurred between 12 and 24 months after rituximab administration. Therefore, it is highly likely that the incidents were not directly related to rituximab. Our study found that rituximab appeared to be very safe for pemphigus during the COVID-19 pandemic. We believe that this was due to the good preparation of our patients before rituximab initiation as well as meticulous care over the following several years by the same medical staff after administration of the drug.

Although there are several publications in the scientific literature on the use of rituximab in pemphigus during the COVID-19 pandemic, most of them refer to concerns about its safety during this particular period. Especially at the beginning of the COVID-19 pandemic, some authors recommended postponing the use of rituximab during this period [37], while others recommended using rather low doses of rituximab in patients with mild-to-moderate pemphigus [38].

In our study, rituximab appeared to be not only safe but also highly effective for our patients during the COVID-19 pandemic as well as in long-term follow-up since more that 80% remain in cCR off therapy. This observation is novel, and has not been published before.

## 5. Conclusions

The conclusions coming from our study are as follows:

1. The current study has proven the high effectiveness and safety of one cycle of rituximab, especially in PF, in long-term follow-up. Therefore, rituximab may be recommended as a first-line treatment in PF; however, further studies on uniform, well-defined groups of patients are needed to support our observations.
2. If patients remain in cCR for at least 36 months, they appear to have a good chance of maintaining long-term cCR and perhaps even a cure.
3. Rituximab has been proven to be very safe and effective during the COVID-19 pandemic, which means that this drug may be a better therapeutic option than other immunosuppressive drugs in similar circumstances.
4. Factors such as age, gender, BMI, the PDAI, and the duration of pemphigus prior to rituximab administration do not appear to influence the achievement of long-term cCR.
5. A direct comparison of remission rates between our study and prior studies is difficult to perform because of different treatment protocols and conditions.
6. Further prospective multicenter studies containing patients on uniform therapeutic protocols are required for a more real assessment of outcomes after rituximab therapy in pemphigus.

## 6. Limitations

The current study was performed in a single dermatological department, the analysis was limited to the clinical and demographic parameters, the subgroup of PF was small, and the nature of the study was retrospective.

Immunological parameters will be discussed in a separate manuscript.

## Figures and Tables

**Table 1:** Clinical characteristics of patients included in study.

Diagnosis	PV	PF
Number of patients	22	6
Age, years	51.5 (30–68)	54.0 (40–67)
Sex		
Female	13 (59.1%)	4 (67%)
Male	9 (40.9%)	2 (33%)
Disease duration before rituximab (months)	28.0 (3–181)	60.0 (7–108)
PDAI at rituximab administration	15.9 (2–66)	22.5 (8–39)
BMI at rituximab administration	31.4 (19.1–36.9)	28.8 (21.1–34.6)
Rituximab administration During COVID-19 pandemic	13	4

Legend: Median (min–max), PDAI—pemphigus disease activity index, BMI—body mass index, and COVID-19—infection caused by SARS-CoV-2.

**Table 2:** Outcomes after first administration of rituximab.

Patients	PV	PF
Number	22	6
Time to remission (months)	1.75 (0–8)	5.5 (1–9)
Duration of remission lasting at least 12 months	17 (77.3%)	5 (83.3%)
18 months	14 (63.6%)	5 (83.3%)
24 months	10 (45.5%)	5 (83.3%)
36 months	10 (45.5%)	5 (83.3%)
Adverse effects		
Infusion-related		
Common	4 (18.2%)	2 (33.3%)
Serious/relevant	0 (0%)	0 (0%)
Follow-up		
Minor	5 (22.7%)	2 (33.3%)
Relevant	2 (9.1%)	1 (16.7%)

**Table 3:** Risk factors for maintaining at least 12-month-long remission in patients with PV, analyzed by univariate logistic regression.

	PV			PV + PF		
Factor	OR	95% CI	*p*	OR	95% CI	*p*
Gender	0.69	0.12–3.78	0.67	0.909	0.39–2.14	0.83
BMI > 25	0.39	0.05–2.77	0.350	0.44	0.07–2.80	0.38
Duration of disease	1.00	0.98–1.02	0.89	1.00	0.98–1.02	0.75
Age at Rtx therapy	0.97	0.90–1.04	0.36	0.98	0.91–1.04	0.49
PDAI > 35	1.78	0.13–23.5	0.66	2.00	0.27–14.59	0.49

OR—odds ratio; 95% CI—95% confidence interval.

**Table 4:** Risk factors for maintaining at least 12-month-long remission in patients with PV, analyzed by multivariate logistic regression.

	PV			PV + PF		
Factor	OR	95% CI	*p*	OR	95% CI	*p*
Gender	0.67	0.06–7.79	0.761	0.94	0.10–8.42	0.953
BMI > 25	4.28	0.26–70.25	0.309	6.09	0.43–85.99	0.181
Duration of disease	1.01	0.98–1.05	0.383	1.01	0.98–1.03	0.534
Age at Rtx therapy	0.91	0.80–1.03	0.130	0.91	0.81–1.02	0.093
PDAI > 35	0.50	0.01–13.72	0.681	0.46	0.03–7.87	0.595

OR—odds ratio; 95% CI—95% confidence interval.

## Data Availability

Additional data are available upon reasonable request, after anonymization, due to privacy restrictions.

## Abbreviations

The following abbreviations are used in this manuscript:

PV	Pemphigus vulgaris
PF	Pemphigus foliaceus
cCR	Complete clinical remission
BMI	Body mass index
DIF	Direct immunofluorescence
IIF	Indirect immunofluorescence
PDAI	Pemphigus Disease Activity Index
COVID-19	Infection caused by SARS-CoV-2
